# Pervaporation Zeolite-Based Composite Membranes for Solvent Separations

**DOI:** 10.3390/molecules26051242

**Published:** 2021-02-25

**Authors:** Roberto Castro-Muñoz, Grzegorz Boczkaj

**Affiliations:** 1 Department of Process Engineering and Chemical Technology, Faculty of Chemistry, Gdansk University of Technology, 11/12 Narutowicza St., 80-233 Gdansk, Poland; grzegorz.boczkaj@pg.edu.pl; 2 Tecnologico de Monterrey, Campus Toluca, Avenida Eduardo Monroy Cárdenas 2000, San Antonio Buenavista, Toluca de Lerdo 50110, Mexico

**Keywords:** pervaporation, zeolite membranes, azeotropic mixtures, volatile organic compounds

## Abstract

Thanks to their well-defined molecular sieving and stability, zeolites have been proposed in selective membrane separations, such as gas separation and pervaporation. For instance, the incorporation of zeolites into polymer phases to generate composite (or mixed matrix) membranes revealed important advances in pervaporation. Therefore, the goal of this review is to compile and elucidate the latest advances (over the last 2–3 years) of zeolite applications in pervaporation membranes either combining zeolites or polymers. Here, particular emphasis has been focused on relevant insights and findings in using zeolites in pervaporative azeotropic separations and specific aided applications, together with novel concepts of membranes. A brief background of the pervaporation process is also given. According to the findings of this review, we provide future perspectives and recommendations for new researchers in the field.

## 1. Introduction

Conventional distillation is the traditional process used for the separation of azeotropic solvent mixtures; however, its multiple drawbacks (high cost, high energy consumption, low efficiency, and secondary pollution) have encouraged the exploration of new emerging technologies that can release a better energy-efficient relationship. At this point, specific membrane technologies, such as pervaporation (PV), have shown their great potential in selective gas and solvent separations with lower energy demand [1,2]. PV, which is identified as a promising alternative for evaporation, drying and distillation, is the combination of permeation and evaporation processes being able to break the azeotropic point of many solvent mixtures [3]. To date, PV has been successfully applied in the separation of plenty of binary water-organic (water-ethanol; isopropanol-water, water–butanol, water–acetic acid, etc.) [4,5,6], organic-water (ethanol-water, isopropanol-water, butanol-water, furfural-water, ethylene dichloride-water, etc.) [7,8,9], and organic-organic (methanol- methyl *tert-*butyl ether, acetone-butanol, benzene-cyclohexane, etc.) [10,11,12] azeotropic mixtures, among others. This membrane technique is recognized as highly selective; unfortunately, its primary disadvantage deals with its low permeation rates related to the properties of the membrane materials used. Initially, polymers have been the pioneering materials used to manufacture PV membranes; therefore, chemical engineers are continuously looking for new membrane materials and new concepts of membranes that may overcome the limitation of polymer membranes. For instance, several inorganic materials have been proposed for the role of selective separation in PV technology, including metal-organic frameworks, graphene-based materials, silicas, covalent-organic frameworks, and zeolites [13,14,15,16]. The latter materials have multiple advantages compared with other inorganic materials, as reported in Table 1. Their well-established molecular sieving and high hydrothermal stability (depending on their Si/Al ratios) make zeolites potential candidates in PV separations, depending on the zeolite structure with superior separation efficiency over polymeric membranes [1,17]. Thanks to these promising features, NaA zeolite membranes have been the only membranes to be commercialized and proposed for large-scale purposes [18,19,20]. In general, zeolites are crystalline aluminosilicate materials composed of primary elements, including potassium, sodium, magnesium, and calcium [21,22]. Zeolites possess an inorganic three-dimensional structure and a four-connected framework of AlO_4_ and SiO_4_ tetrahedra linked to each other by sharing an oxygen ion. Due to the presence of AlO_4_ tetrahedra, the frameworks generally display a negative charge that can be compensated by alkali (Na or K) or earth-alkali (Mg or Ca) cations and water molecules. According to the literature and official databases (http://www.iza-structure.org/databases, accessed on 19 February 2021), there are more than 250 zeolites that have been synthesized, including zeolites X, Y, A, and Zeolite Socony Mobil–5 (ZSM-5). Zeolites are well-identified excellent molecular sieving material (adsorbent) for selective separation. The classification of zeolites comprises small pore zeolites with 8-ring apertures (0.3-0.45 nm), medium pore zeolites with 10-ring apertures (0.45-0.6 nm), large pore zeolites with 12-ring apertures (0.6-0.8 nm), and extralarge pore zeolites with apertures of more than 12 rings. ZSM-5 belongs to the medium pore zeolites with pore apertures in the range 0.51-0.56 nm [21].

When dealing with membrane manufacture, zeolites tend to offer great flexibility since they have been utilized in different ways, including in pristine form, combined with polymers and other inorganic phases to tailor mixed matrix membranes (or composites), supported on stable membrane supports [23,24,25]. To date, various zeolites, including zeolite A, mordenite, and merlinoite, have been implemented in membrane synthesis [26,27], exhibiting interesting water permeability when used in PV separations. At this point, the water transport in zeolites is directly dependent on the Si/Al ratio, since such a relationship influences the final adsorption of the hydrophilic sites of zeolites [27]. Such an important basis has led researchers to explore zeolites in different concepts of membranes for different types of PV applications. Therefore, the target of this work is to review the latest advances in various manufacturing concepts of zeolite-based membranes investigating their separation performance for PV. Here, the recent breakthroughs were reviewed and analyzed, highlighting the relevant findings and results in terms of membrane transport towards solvents. For new readers in the field, a summarized background and basic concepts of PV unit operations are also introduced. To finalize, conclusions, future perspectives, and recommendations are stated based on the current insights of the literature.

## 2. Fundamental Aspects of Pervaporation

**Figure 1:**
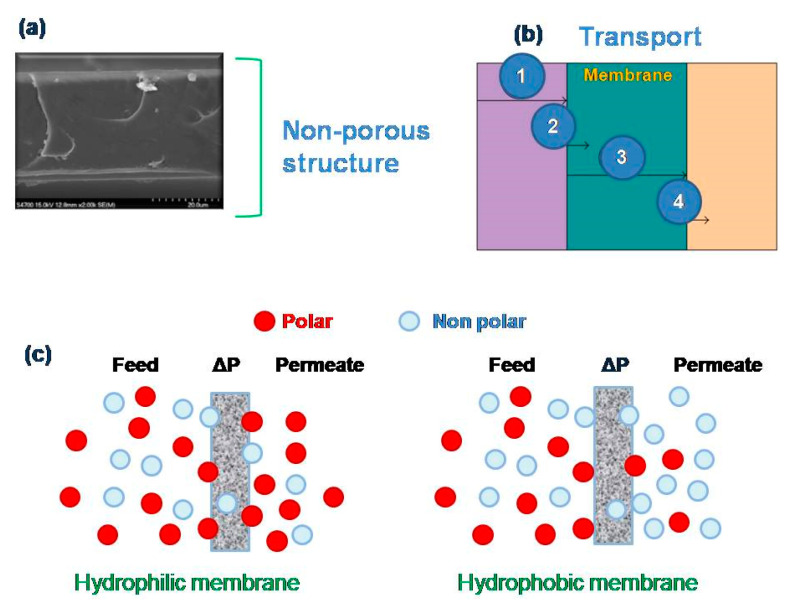
Representation of a typical structure of a dense membrane: (**a**) its transport mechanism (**b**) and the molecule transport in hydrophilic and hydrophobic membranes. (**c**) hydrophilic membranes have a higher affinity to the most polar compounds, while hydrophobic membranes have a higher affinity to the less polar compounds (including nonpolar).

As stated previously, the PV technique is a unit membrane operation utilized for the separation of wide types of azeotropic solvent mixtures [28]. This particular membrane technique uses membranes that have a non-porous (or identified as dense) structure, as depicted in Figure 1a. The mass transport over non-porous membranes is established by the so-called solution diffusion mechanism implying three stages, namely, (i) the diffusion of the mixture components through the liquid boundary layer to the active membrane surface; (ii) the sorption/diffusion of mixture components into the membrane; (iii) transport across the membrane structure; and (iv) the diffusion/desorption of components through the vapor phase boundary layer into the permeate side (Figure 1b) [29]. Mass transport is a function of the chemical potential, physicochemical properties of the permeating species, and their concentration on both feed bulk and permeate sides. In the case of the physicochemical properties of the species, the polarity of the molecules is a key fundamental when transported in hydrophilic and hydrophobic PV membranes; for instance, hydrophilic membranes have a higher affinity to the most polar compounds, while hydrophobic membranes have a higher affinity to the less polar compounds (including nonpolar), as illustrated in Figure 1c. According to Equation (1), the permeability (P) in a PV membrane is the product of solubility(S) and diffusivity (D) parameters of the mixture compounds. The S parameter is independent of the concentration of the compounds, while the D parameter depends on the geometry of the compounds (e.g., an increase in molecular size results in a diffusion decrease) and concentration [30].

Permeability (P) = Solubility (S) * Diffusivity (D)(1)

Regarding the effectiveness and performance of a PV process, the permeate flux (J) and separation factor (β) are the primary parameters to be determined. The calculation of J for the component *i* (J_i_) comprises the quantification of its mass (m_i_), which is able to permeate through a certain active area (A_m_) and time (t), as denoted in Equation (2). The β factor, which estimates the separation efficiency of a PV process, is calculated by knowing the concentration of the components *i* and *j* in feed and permeate side (see Equation (3)).

(2) $$J_{i}=\frac{m_{i}}{A_{m}\;\cdot \;t}$$

(3) $$\beta_{i}={\frac{C_{i}}{C_{j}}}_{permeate}/{\frac{C_{i}}{C_{j}}}_{feed}$$

Apart from J and β, the performance of a PV process can also be expressed in terms of the pervaporation separation index (PSI), which determines the overall performance of a PV operation [31]. It represents a direct relationship between J and β parameters, and it can be calculated as follows:

(4) PSI=J ·β

In this expression, the PSI can be large if the membrane has a high flux even when β = 1; thus, the definition of PSI parameter can be modified as:

(5) PSI=J ·(β−1)

## 3. Latest Insights into Zeolite-Based Membranes for Pervaporation

### 3.1. Dehydration of Organics

Among the core applications of PV technology in separating azeotropic mixtures relies on the dehydration of organics. In other words, it implies the removal of traces of water from different types of organic solvents at azeotropic concentration. At this point, according to their hydrophilic nature, zeolites represent promising membrane materials for the selective separation of water. For instance, Cao et al. [32] fabricated high-permeation NaA zeolite membranes (thickness of 3.7–4.5 μm) on alumina hollow fiber, displaying unprecedented performance in ethanol dehydration with flux of 19.7 kg m^−2^ h^−1^ and β of over 80,000. The high permeation rate was a result of the high operating temperature used, together with the membrane concept, since hollow fibers are well recognized for their high productivities in terms of flux due to the larger effective surface area to volume ratio [33]. Additionally, this PV process using NaA fiber demonstrated a temperature dependence according to the Arrhenius analysis. In a different study, hollow fibers based on modernite zeolite also showed high performance when dehydrating ethanol, acetic acid, and isopropanol (IPA) (see Table 2) [34]. Compared to Cao’s findings, Chen and co-workers [34] obtained lower flux and β, but they are still competitive in the field of pervaporation. Moreover, modernite-based membranes were able to be operated at a high content of water, although specific zeolites (such as LTA) have shown poor stability in water-enriched mixtures. Generally, low stability is observed at a low Si/Al ratio [35], but in the case of modernite, presenting low Si/Al ratios, offered good stability in operation over 120 h (at 100 °C) for the separation of water-ethanol (50 wt.% concentration) [34]. In a pilot-scale study, NaA zeolite membranes supported on stainless-steel tubes were synthesized by Gui et al. [36], who subsequently evaluated their performance in separating water from ethanol. Here, the authors also reported a high flux (ca. 8.2 kg m^−2^ h^−1^) and separation efficiency (ca. 11,000). Apart from the excellent PV performance, the membranes were operated over a month, providing reliable stability for possible large-scale operation. Similar impressive β values ranging from 10,000 to 100,0000 were documented by Liu et al. [37] during the dehydration of several alcohols, such as methanol, ethanol, and IPA, implementing Lynde type A(LTA) zeolite membranes in the PV process. The obtained permeate samples contained a water concentration as high as 99.99 wt.%.

**Figure 2:**
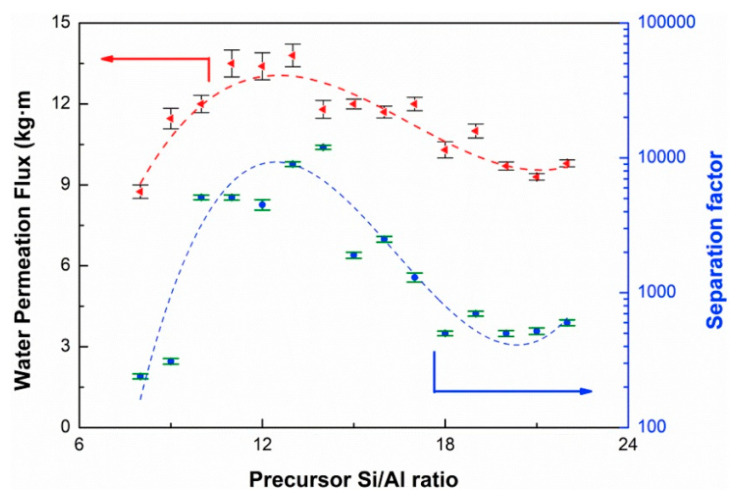
Effect of precursor Si/Al ratio of CHA zeolite membranes for ethanol dehydration (10 wt.% water, at 75 °C). Reproduced with permission from [39]; published by Elsevier, 2019. License number 5010730294396.

An important factor during the synthesis of zeolites is the Si/Al ratio, since such a relationship dictates the resulting features (e.g., hydrophilicity) of the zeolite membranes and thus influences their separation yield. This becomes important due to the fact that water transport depends on the selective adsorption into the hydrophilic sites of the zeolite [69]. For instance, Jiang et al. [39] evidenced the role of the Si/Al ratio in the performance of a CHA zeolite hollow fiber membrane, as represented in Figure 2. At a low Si/Al ratio of 2.7, the membranes possessing flake-like grains exhibited higher J with low selectivity, but the β increased over 10,000 when the Si/Al ratio was higher than 2.9. The investigation reported that both the hydrothermal and acid stabilities of the zeolite fibers were also dependent on the Si/Al ratio over 250 h of testing. The Si/Al ratio also impacted membrane structure, e.g., when the precursor Si/Al ratio increased from 11 to 13, the membrane structure changed morphologically from flake-like grains to block-shaped crystals, resulting in an increase in β from 5000 to 9000; surprisingly, the increment in the precursor Si/Al ratio to 14 resulted in a J decrease of about 12 kg m^−2^ h^−1^ [39]. It is worth mentioning that the increase in the Al amount in zeolites provokes higher hydrophilicity. Unfortunately, most of the zeolite membranes with a low-silica content present low acid-stability due to excessive dealumination. This factor makes their use in acidic solutions difficult. On the contrary, a high silica content, such as silicalite-1, provides more stability due to the fact that the membrane tends to be hydrophobic. To date, important efforts have been devoted to using the excellent performance of this zeolite even under acidic conditions [70].

NaA zeolite lacks good performance and stability in an acidic environment. Based on these limitations, the research community is exploring new strategies that may confer enhanced properties to this zeolite, allowing its application to be extended. For example, Li et al. [40] proposed the impregnation of a positively charged polyelectrolyte (PEC) complex based on chitosan and sodium poly (vinyl sulfonate) onto a NaA zeolite surface to generate PEC/NaA composite membranes. After full characterization, the authors stated that the pristine zeolite NaA membrane showed severe damage in terms of morphology and crystal structure in contact with acid, while in turn, the PEC/NaA composite remained unchanged. When testing under acidic conditions (pH = 3) for ethanol dehydration, the flux was about 0.875 kg m^−2^ h^−1^, in which the permeate was around 99.8 wt.% enriched in water. Finally, these complex membranes showed acceptable acid stability over 200 h thanks to the barrier effect of the PEC layer and the protonation ability of amino groups in PEC. In a different investigation, Qiu et al. [44] documented the long-term stability (over 35 h) of choline chloride templated CHA zeolite membranes, which had a relatively higher Si/Al ratio (between 3.6 and 3.9) as expected, the authors noted that the Si/Al ratio and choline chloride greatly influenced the PV performance when dehydrating ethanol and acetic acid. For instance, the synthesized CHA membranes exhibited a permeation between 4.7 and 5.6 kg m^−2^ h^−1^ and impressive separation efficiency (β over 2000) in water/ethanol binary separations. According to the Arrhenius relationship, the ethanol and IPA permeations were apparently much higher than those of water, which were 53.3 ± 2.6 kJ mol^−1^ and 52.4 ± 1.2 kJ mol^−1^, respectively, indicating that the transport of water was easier compared with ethanol and IPA [44]. When dehydrating acetic acid, these membranes also offered a high permeation rate, which decreased from 4.6 to 2.5 kg m^−2^ h^−1^ during the first 15 h, attributed to the adsorption of acetic acid together with dissolved amorphous aluminosilicate blocked in the intercrystalline boundaries, and after such a period, the permeation was maintained constant over 35 h. Finally, the resulting permeate contained around 98 wt.% water. Kursun [56] also presented a study reporting high separation efficiency (β = 2690) for IPA dehydration utilizing NaY-loaded PVA membranes; however, a very low permeation (ca. 0.005 kg m^−2^ h^−1^) was observed in such membranes. In general, when an inorganic filler is incorporated in polymer phases, a motion restriction of the polymer chains takes place and thus an increase in rigidity of the resulting membrane. Additionally, the free volume tends to decrease and negatively influence the diffusivity of molecules through the membranes obtaining less flux. At this point, the low permeation in NaY-loaded PVA membranes is a result of such contributions [56].

Thanks to the great hydrophilic nature and acid-resistance of chabazite zeolite, Wu et al. [66] prepared chabazite hollow fibers via a two-stage varying temperature hydrothermal protocol. Here, a crystallization condition of 150 °C (for 10 h) was used at the first stage, followed by 100 °C (for 5 h) at the second stage. During the dewatering of ethanol (10 wt.% water in feed, at 75 °C), these zeolite membranes offered a stable flux (6.25 kg m^−2^ h^−1^) and separation (β = 1950). Intentionally, the researchers varied the water concentration (30 wt.%) in feed, still obtaining a stable operation over 240 h with a high flux (ca. 10 kg m^−2^ h^−1^) and separation efficiency (ca. 1200).

Chemical modification is a current research pathway aimed at improving the physicochemical and separation features of zeolites [71]. Thanks to the change of sodium ions by copper ions on LTA zeolite membranes, an increase in pore size caused an improved permeation as demonstrated by Xu et al. [48]. Na-LTA and Cu-LTA membranes exhibited a water flux increase as a function of temperature, e.g., the water flux of the Na-LTA and Cu- LTA membrane was about 0.32 and 0.59 kg m^−2^ h^−1^ (10 wt.% water, at 30 °C), which increased to 1.65 and 3.52 kg m^−2^ h^−1^ at 75 °C, respectively. In the range of 30–75 °C, water fluxes across the Cu-LTA membrane were higher than those of the Na-LTA zeolite membrane. According to the author’s findings, such enhancement was related to the increase in pore size that provoked a decrease in mass transfer resistance, and therefore, an increase in water flux [48]. Comparable permeation rate (ca. 4.2 kg m^−2^ h^−1^) but higher separation efficiency (β exceeding 10,000) was reported by Gao et al. [54], who applied a stainless-steel hollow fiber supported NaA zeolite membrane to dehydrate ethanol under a similar condition to that of Xu et al. [48].

**Figure 3:**
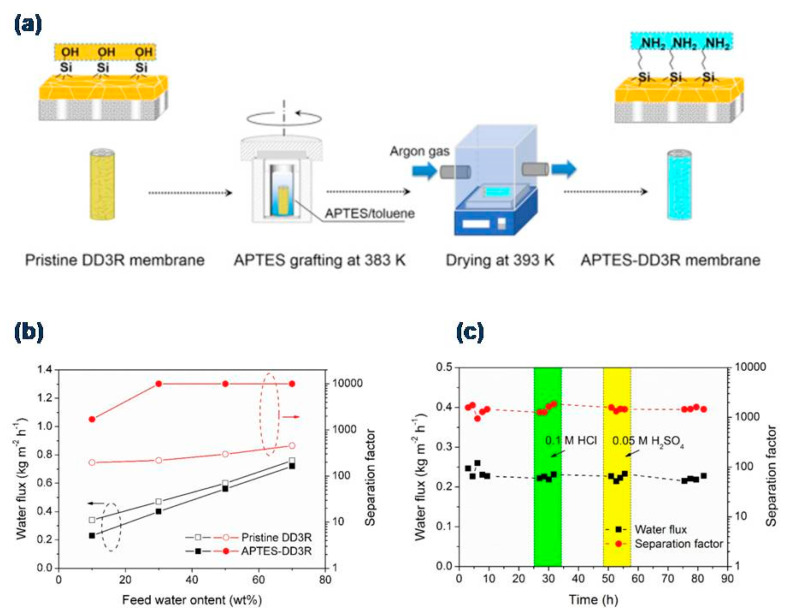
3-aminopropyltriethoxysilane (APTES) grafted deca-dodecasil 3 rhombohedral (DD3R) membrane proposed by Zhang et al. for acetic acid dehydration (10 wt.% water, 75 °C, 200 Pa). Reproduced with permission from [50]; published by Elsevier, 2019. License number 5010730591021.

Zhang et al. [50] used 3-aminopropyltriethoxysilane (APTES) to graft the surface of hollow fiber supported deca-dodecasil 3-rhombohedral (DD3R) membrane (see Figure 3a), aiming at the improvement of its surface hydrophilicity and stability in acidic pervaporation. By a combined effect of facilitated water adsorption on the surface and an intrinsic molecular-sieving mechanism of DD3R zeolitic pores, the APTES-DD3R membranes released an enhanced β of 1700, a higher value than 196 in the pure DD3R membrane when separating 10 wt.% water/acetic acid mixture (at 75 °C). These membranes definitely showed an enhanced PV yield compared with the unmodified membranes, as illustrated in Figure 3b. Additionally, the membranes also possessed excellent stability (more than 80 h, see Figure 3c) in the presence of strong inorganic acids (HCl and H_2_SO_4_). Preliminarily, Li and co-workers also investigated the pervaporative separation of water-acetic solutions by means of zeolite-based membranes [62,63]. In these cases, ZSM-5 and mordernite membranes acted as water selective barriers displaying β and J values of about 3200 and 0.98 kg m^−2^ h^−1^, respectively, for the ZSM-5 membrane [62]. However, mordernite membranes had values of 1200 and 0.97 kg m^−2^ h^−1^, respectively, when dehydrating acetic acid in a scaled-up operation [63]. In addition to their good performance, both membranes had high reproducibility, presenting a promising large-scale application for dehydration in acidic conditions.

### 3.2. Separation of Organics from Diluted Azeoptropic Mixtures

The use of PV does not always deal with the separation of water from organics. By smartly selecting the membranes, the separation of mixtures containing organic-water and organic-organic phases can also be successfully performed. Alomair and Alqaheem [38] utilized ZSM-5 membranes for the separation of p-xylene from its bulkier m-xylene and o-xylene. Since xylene isomers, such as p-xylene, m-xylene, and o-xylene, display very similar boiling point values, this makes their extraction difficult by means of traditional methods [72]. Therefore, the authors proposed their selective split by pervaporative separation, revealing a β value of about 1.46 and flux of 0.12 kg m^−2^ h^−1^. Although the performance was not the best in comparison with other zeolite membranes (see Table 3), these membranes were able to exhibit a higher performance by changing the feed concentration, e.g., β of 5.35 and flux of 0.076 kg m^−2^ h^−1^ (at 25 °C, 95 wt.% o-xylene in feed).

Towards the direct extraction of organic compounds from diluted aqueous systems, Ma et al. [42] showed that MFI zeolite membranes on super hydrophobic 1H,1H,2H,2H-perfluoroalkyltriethoxysilanes (POTS) supports exhibited the ability to remove ethanol from water (5.0 wt.% ethanol in feed, at 75 °C). The study reported that the MFI zeolite membrane showed high selectivity for ethanol (β of 103) together with high J (ca. 2.56 kg m^−2^ h^−1^) in this kind of separation. With such a performance, these membranes are in fact promising candidates to recover bioethanol from fermentation stages. Unlike Ma’s investigation, Ueno et al. obtained low permeation (0.62 kg m^−2^ h^−1^) but higher selectivity (β of 229) for butanol separation using BEA-type zeolite membranes [45]. Aiming at the recovery of n-butanol from diluted aqueous systems, Cheng et al. [53] recently embedded ZSM-5 zeolite into polydimethylsiloxane (PDMS); these resulting mixed matrix membranes (loaded with 40 wt.% ZSM-5) showed an acceptable n-butanol separation (β of 77) and total flux of 0.11 kg m^−2^ h^−1^, in which 0.062 kg m^−2^ h^−1^ corresponded to n-butanol when testing a 1.5 wt.% n-butanol solution (at 47 °C). As a concluding remark, the researchers stated that these composite membranes could reduce the energy consumption for butanol recovery from 1 wt.% butanol by ≈75% to ≈10 MJ kg^−1^ butanol using a pervaporation–distillation process in comparison with distillation.

**Figure 4:**
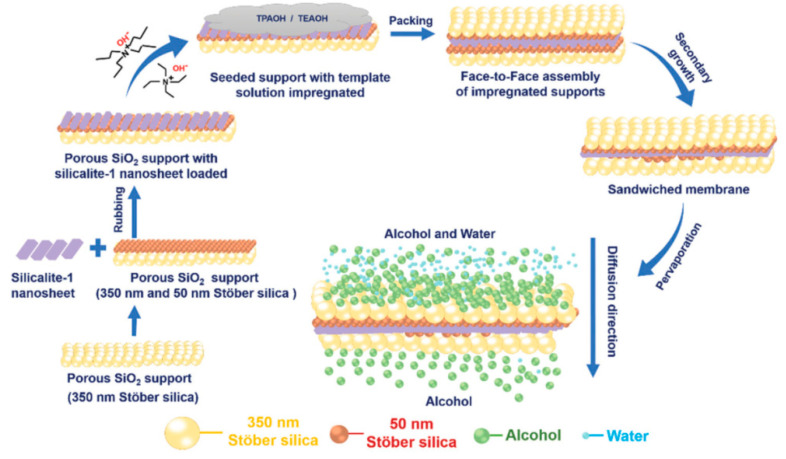
Graphical depiction of the sandwiched silicalite-1 membrane for the recovery of ethanol and n-butanol from water. Reproduced with permission from [59]; published by The Royal Society of Chemistry (RSC), 2020.

A current breakthrough in tailoring new concepts of zeolite-based membranes is that reported by Prof. Tsapatsis [59]. In this work, they designed a sandwiched (SiO_2_)/(silicalite-1)/(SiO_2_) in a 2D confined space, as illustrated in Figure 4. This interesting membrane had a zeolite active layer of ~500 nm, which provided a meaningful PV performance when recovering ethanol and n-butanol from diluted aqueous systems. For example, ethanol and n-butanol separation values of 136 and 113 were acquired, respectively, while the flux values were about 2.3 and 2.2 kg m^−2^ h^−1^, respectively. Such yield is attractive for these applications, since hydrophobic membranes do not generally offer high fluxes. The authors stated that the unprecedented separation performance can be associated with several factors, such as the confined geometry improving intergrowth, by-skipping the calcination stage in the synthesis, diminishing defects, and the usage of a 2D material (such as graphene oxide) for the synthesis of silicalite-1 seeds fostering hydrophobicity in the final membrane [59]. To conclude, this novel membrane also stands out in the ability to operate over 14 days. Comparable high performing membranes were also reported by Mirfendereski and Lin [60], stating a flux of 3 kg m^−2^ h^−1^ and a β value of 160 for ethanol separation via MFI zeolite hollow fiber membranes synthesized by double-layer seeding.

By applying a postsynthesis atmospheric-pressure plasma jet, Tao et al. [65] considerably enhanced the performance of FER zeolite membranes for ethanol dehydration; for example, the β increased from 93 to 377 with the enhancements of the water flux from 0.040 to 0.045 kg m^−2^ h^−1^ (10 wt.% water, 75 °C). According to the authors, the treatment also presented a remarkable influence on the β of MOR membrane, e.g., from 141 to 286. Such enhancements were related to an increase in surface hydrophilicity that inherently promoted the water adsorption and thus increased the driving forces for water diffusion. Moreover, the yield improvement was associated with partial elimination of pinholes according to the air permeation experiments.

In an atypical application, Pan et al. [43] filled hollow monocrystalline silicalite-1 into a Pebax^®^ 2533 polymer to produce hybrid membranes and subsequently conduct pervaporative desulfurization. These composite membranes were strategically fabricated, since the micropores on the silicalite shell enhanced selectivity due to a sieving effect, while its inner cavity promoted the fast diffusion of molecules (e.g., thiophene/n-octane) producing an enhanced flux. At 20 wt.% silicalite loading, the composite membranes demonstrated a flux of 20.6 kg m^−2^ h^−1^ and an enrichment factor of 6.11, which represented an 82% and 23% higher performance compared with the pristine polymer membrane, respectively. In addition to this, the membrane displayed considerable anti-swelling properties and long-term stability operating over 7 days.

In a series of studies, Vatani and co-workers synthesized high performing hydrophobic membranes for the recovery of ethyl acetate from diluted aqueous solutions. Ethyl acetate is typically used as a chemical solvent among different industries for the manufacture of drugs, perfumes, plasticizers, and varnishes, to mention just a few [81,82]. Its synthesis relies on the typical esterification reaction of acetic acid with ethanol [83,84], while its separation at a low concentration from water and ethanol is needed. Initially, mixed matrix membranes based on ZSM-5/poly (ether-block-amide)/polyethersulfone were proposed by Vatani et al. [46], who separated ethyl acetate from aqueous solution; the membrane loaded with 7.5 wt.% ZSM-5 had the best β value of 108 with a permeation rate of about 1.8 kg m^−2^ h^−1^ (5 wt.% ethyl acetate in feed, at 50 °C). The authors also observed an increase in permeation by raising the temperature between 30 and 50 °C associated with the increase in mass transfer driving force. Additionally, the increase in temperature results in a stronger polymer chain motion and consequently less membrane resistance for the permeation of molecules [29]. Concurrently, the authors also developed a new concept of mixed matrix membranes presenting [Hmim][PF6] ionic liquid and ZSM-5 nanoparticles based on poly (ether-block-amide). In sum, the membrane containing 2.5%wt. ionic liquid showed the best separation yield in terms PSI of 51.5 kg m^−2^ h^−1^, corresponding to a flux of 1.03 kg m^−2^ h^−1^ [47].

The removal of water from dimethyl carbonate (DMC) solutions was carried out by Zhou et al. [67]. Here, a large tubular zeolite FAU membrane was implemented in the PV set-up; this particular membrane presented an excellent ability in separating water due to its large pore size (≈0.74 nm) and high hydrophilic profile (associated with the low Si/Al ratio). The flux of this membrane was up to 3.60 with optimal β (>10,000) when treating a 10 wt.% water in DMC mixture, in which the retentate at the end of the operation (over 9 h) contained up to 99 wt.% of DMC. Additionally, the membrane demonstrated an Arrhenius relationship in permeation as a function of operating temperature.

### 3.3. Separation of Organic-Organic Azeoptropic Mixtures

A challenging organic-organic separation concerns the purification of methyl *tert-*butyl ether (MTBE); the importance of this chemical relies on its use as an octane enhancer and an excellent oxygenated fuel additive for gasoline formulation to mitigate air pollution produced by vehicle emissions [85]. MTBE production basically implies the reaction between methanol and isobutylene; additional methanol is typically added to reach a higher yield. Unfortunately, MTBE and excess methanol can form an azeotrope at 14.3 wt.% methanol concentration. In this process, distillation is usually the technique used to break the azeotrope, representing a costly and energy intensive method. Over the last decade, tremendous effort has been made in implementing PV as an alternative to such separation [86]. Of course, zeolites have also been proposed to separate such organic-organic mixture with the need for methanol selective materials. For instance, Li et al. [41] synthesized thin and (h0l)-oriented zeolite Al-beta membranes that had a highly hydrophilic nature (water contact angle of 45°). Such membranes demonstrated their efficiency and acceptable permeation with J and β values of 1.83 kg m^−2^ h^−1^ and 20, respectively. The authors noted that the permeation of methanol and the membrane selectivity were negatively affected when the methanol content was increased, while the permeation of MTBE varied slightly. According to the authors, this could be associated with a possible crowding effect of methanol diffusion through zeolitic pores [87].

More recently, Zhu et al. [51] evaluated the effect of fluoride-presenting precursors for the manufacture of NaY zeolite membranes for separating ethanol/ethyl *tert*-butyl ether. Typically, the separation yield was dependent on the operating temperature and the feed composition, showing a constant β value (ca. 1100) and flux (ca. 1.30 kg m^−2^ h^−1^) (20 wt.% ethanol in feed, at 60 °C). Interestingly, when the feed contained 10 wt.% ethanol, the flux still exhibited a temperature dependency, while the β was independent, which may be associated with the kinetic diameter difference of ethanol (≈0.44 nm) and ethyl *tert*-butyl ether (≈0.62 nm) molecules. Additionally, this zeolite membrane has proved its reliability in performing long-term operations of over 80 h.

### 3.4. Zeolite-Based Membrane-Aided Specific PV Applications

To date, zeolite membranes implemented in PV processes have shown their ability to efficiently separate different types of water-organic, organic-water, and organic-organic mixtures; nonetheless, the versatility of PV has gone further in assisting specific applications. For example, water scarcity is among the worldwide challenging issues, where the production of drinking water via seawater desalination has become an alternative. At this point, PV technology has been explored in such a field, releasing interesting insights. Considering their hydrophilic properties and facilitated water transport, zeolites are excellent candidates for the purification of water and hindrance of salt permeation. For instance, nanocomposite membranes based on AEL zeolite and polyamide, with a ≈400 nm thickness, displayed high water transport and NaCl rejection in the desalination of low (ca. 2 g L^−1^ NaCl) and high (ca. 36 g L^−1^ NaCl) salt aqueous mixtures [88]. Regardless of the low operating temperature (at 25 °C), the nanocomposite membranes reported a high flux of about 4.3 and 3.3 kg m^−2^ h^−1^ for the solutions containing 2 and 36 g L^−1^ NaCl, respectively, while the salt rejection was always maintained over 99.9% independently of the salt concentration (see Table 4). In this work, the authors stated that such exceptional water permeation could be attributed to the intermediate AEL nanosheet layer with microporous nature reducing the water diffusion path length, together with the higher effective surface area of the polyamide layer surface [89] and the application of alumina hollow fibers as a substrate for the polyamide membrane, allowing enhanced water fluxes to be reached due to the hydrophilicity of alumina. Moreover, these nanocomposites were able to offer a stable operation in a longer term operation for 150 h. Wang et al. [90] were also in agreement with Korde’s study for the use of alumina as a support for the synthesis of NaA zeolite membranes. The authors observed high water permeation (≈9.58 kg m^−2^ h^−1^) with a complete salt rejection (~99.9%) in the desalination of 3.5 g L^−1^ NaCl solution (at 75 °C). There is a wide difference in permeation rates among both studies due to the difference in operating temperature. Interestingly, Wang and co-authors [90] extended the application of NaA zeolite membranes for the desalination of other types of salt mixtures, such as KCl, CaCl_2_, and MgCl_2_ solutions. The water fluxes were around 8.62, 9.35, and 8.69 kg m^−2^ h^−1^, respectively, along with high salt retention (≈99.9%). However, the authors noticed a water flux reduction when using such salts instead of NaCl; this was associated with four possible phenomena: (i) differences in ion particles causing a different driving force in PV desalination; (ii) ion exchange (K^+^, Ca^2+^, Mg^2+^) could occur on the zeolite membranes due to a difference in pore size; (iii) pore-blocking due to ion deposition; and (iv) different electrostatic interactions between ions and water.

PV can also benefit specific reactions to achieve a higher conversion of reactants. As an example, the water is commonly formed as a by-product within the esterification reaction, which is indeed undesired, since it provokes the hydrolysis of the ester when a thermodynamic equilibrium is reached. To surpass such equilibrium conversion, the incorporation of additional alcohol is the pathway of reaching higher yields, requiring a further separation of the residual alcohol. As a second option, simultaneous water removal, once the esterification reaction takes place, is a preferred method; herein, PV can be involved in situ and ex situ modes. Lv et al. [91], for instance, employed PV to shift the reaction equilibrium of esterification for biodiesel production. At this point, a NaA zeolite membrane was proposed for the simultaneous water elimination from the reactor; free fatty acid conversion rates were found as high as 99% using PV technology (ethanol: oil molar ratio = 15:1, catalyst dosage = 40 wt.%, temperature = 78 °C, over 7 h). This enhancement was expected, since NaA zeolite possesses pore openings of about 0.41 nm, in which smaller molecules, e.g., water with a size of 0.26 nm, are able to pass through, blocking ethanol permeation with a size of 0.44 nm.

The direct separation of organics from the fermentation process can be performed via hydrophobic zeolite membranes. Experimentally, Wu et al. [92] evaluated the performance of the hydrophobic MFI membrane with the recovery of butanol from acetone-butanol-ethanol (ABE) solution. The membrane had butanol selective properties (β = 14) with a representative permeation of 0.12 kg m^−2^ h^−1^, also proving a long-term operation ability over 30 h. This membrane represents a new method of recovering bio-butanol in a more efficient manner from complex fermentation systems.

In different investigations, Zeng et al. proposed NaA zeolite membranes for the extraction of ethanol and sodium pyruvate from waste centrifugal mother liquid [93] and assisting the manufacturing process for lithium-ion battery [94]. In the first study, a low-temperature PV process for water removal was used, and a second staged implying crystallization, followed by filtration to collect ethanol and crude sodium pyruvate, was designed. The NaA zeolite membrane treated ethanol/water/sodium pyruvate mixtures, showing an efficient water/ethanol separation factor (>10,000) under different operating conditions [93], but the water flux increased as a function of temperature due to the increase in water fugacity in the feed, significantly improving the driving force in PV. However, in the second study, NaA zeolite membranes acted as a tool for dehydrating recovered N-methyl pyrrolidone (NMP) within the industries of lithium battery production. After the separation evaluation, the PV unit showed a total flux higher than 2 kg m^−2^ h^−1^ and β values between 500 and 1700 depending on the water concentration in feed, vacuum pressure, and temperature; finally, the resulting NMP retentate presented a concentration of at least 99.9% NMP with minimal quantities of water (less than 105 ppm) [94].

Specific zeolites have been also proposed as a catalytic agent during chemical reactions; this is the case for Ti-MWW zeolite, which is a titanium silicalite with double independent 10-ring channel networks [95]. According to its particular framework, this zeolite presents better catalytic activity than titanosilicate-1. Based on such evidence, Zhu et al. [96] synthesized and later applied Ti-MWW zeolite into a pervaporation membrane reactor (PVMR) for phenol hydroxylation. According to the author’s insights, the zeolite membrane demonstrated a good catalytic yield for phenol hydroxylation under pervaporation temperature at 50 °C, exhibiting a phenol conversion and dihydroxybenzene selectivity of about 22.9% and 98.3%, respectively.

## 4. Concluding Remarks

By exploring the latest literature data (over the last 2-3 years), zeolites have demonstrated their ability in separating various types of azeotropic mixtures by means of pervaporation, in which the dehydration of organics (including alcohols, acids, and ethers) has been the most investigated field due to the preferential water transport of hydrophilic zeolites, while the shiftiness of the Si/Al ratio allows hydrophobic zeolite membranes suitable for the recovery of organics (esters, alcohols, aromatic hydrocarbons, etc.) to be obtained from diluted aqueous and organic systems. Through all these applications, zeolites possess the features to synthesize different concepts of pervaporation membranes, such as mixed matrix membranes and nanocomposites.

To date, the low stability of zeolites in acidic conditions has been identified as the main drawback of these materials for extending their application at large scales and to other separations. Therefore, scientists are hardly developing new strategies to provide enhanced acid-stability to zeolites; in addition to modifying the Si/Al ratio [39], the use of different precursors of zeolite synthesis [44], impregnation [40], and grafting of chemical agents [50] are among the alternative protocols to confer enhanced stability in zeolites. To some extent, it is likely that researchers will be continuously developing new chemical modification techniques of zeolites; however, the performance assessment of zeolites membranes in a long-term operation may be required.

Due to the distinctive frameworks of zeolites, the implementation of zeolites also relies on specific purposes, such as water purification and desalination, membrane-aided reactions, as well as a catalytic agent. Here, due to their excellent hydrothermal stability, zeolites will be a scope of study in the coming years, since they have displayed exceptional water permeation (between 3.3 and 9.5 kg m^−2^ h^−1^) together with almost the complete removal of salt (≈99.9%) in seawater desalination. Thus, it is quite possible that chemical engineers will be focused on designing new types of zeolite membranes that may provide higher permeation rates, making them competitive against other established membrane techniques.

## Figures and Tables

**Table 1:** Characteristics of materials used in membrane separations.

Zeolites	Metal-Organic Frameworks	Silicas	Carbon Molecular Sieves
Defined pore size	Coordinative bonds	Alter the molecular packing of polymer chains	High affinity to glassy polymers
High temperature stability	Flexibility in molecular sieving	Increase the free volume of polymers	High adsorptive capacity
High stability in water presence	Cations interconnected by organic anions	Low permeability of nonporous silica	Well-defined molecular sieving
Limitation in chemical modification	Rather flexible and dynamic frameworks	Weak interaction among silica-polymer	Great potential in MMMs preparation
Pore size crystallographically controlled	Soft structure/flexible pore size	Generate interfacial voids	Goods adhesion at interface
Potential as supported thin film	Low hydrothermal stability	Suitable for chemical modification (silane coupling)	High productivity/excellent separation
Create dense structures	Suitable for chemical modification and blending		Wide opening with constricted apertures
Well-defined molecular sieving	Great potential for thin structures		
Acceptable sorption and diffusion properties	Offer accessible open metals		
Good mechanical stability			

**Table 2:** Recent studies in applying zeolite-based composites for separation of azeotropic mixtures.

Membrane	Azeotropic Mixture	Operating Parameters	Flux (kg m^−2^ h^−1^)	Separation Factor (β)	PSI	Reference
NaA hollow fiber	Water/ethanol	10 wt.% water, 75 °C	19.7	>80,000	157,600	[32]
Modernite hollow fiber	Water/ethanol	10 wt.% water, 75 °C, <200 Pa	1.01	4684	4730	[34]
	Water/IPA	10 wt.% water, 75 °C, <200 Pa	1.45	6963	10,096	
	Water/acetic acid	10 wt.% water, 75 °C, <200 Pa	0.47	2150	1010	
ZSM-5−carbon	p-xylene/o-xylene	50 wt.% water, 25 °C, 8 Pa	0.12	1.45	0.174	[38]
NaA supported stainless-steel	Water/ethanol	10 wt.% water, 75 °C	8.28	11,000	91,080	[36]
CHA hollow fiber	Water/ethanol	10 wt.% water, 75 °C, 200 Pa	12.0*	10,000	120,000	[39]
PEC/NaA composite	Water/ethanol	10 wt.% water, 45 °C, 100 Pa	0.87	-	-	[40]
(h0l)-oriented zeolite Al-beta membranes	Methanol/MTBE	20 wt.% methanol, 50 °C, 133 Pa	1.83	20.3	37.1	[41]
MFI zeolite membranes supported onPOTS	Ethanol/water	5 wt.% ethanol, 75 °C	2.56	103	263.6	[42]
Hollow monocrystalline silicalite-1-filled Pebax	Thiophene/n-octane removal from water	500 ppm sulfur content, 60 °C, 500 Pa	20.6	6	123.6	[43]
Choline chloride templated CHA zeolite membranes	Water/ethanol	10 wt.% water, 75 °C, 200 Pa	4.7	>2000	9400	[44]
	Water/IPA	10 wt.% water, 75 °C, 200 Pa	5.6	>2000	11,200	
BEA-type zeolite membranes	Butanol/water	1 wt.% butanol, 45 °C, 200 Pa	0.62	229	141.98	[45]
ZSM-5/poly (ether-block-amide)/PES	Ethyl acetate/water	5 wt.% ethyl acetate, 50 °C	1.89	108	204.12	[46]
[Hmim][PF6] ionic liquid/ZSM/ poly (ether-block-amide).	Ethyl acetate/water	5 wt.% ethyl acetate, 50 °C	1.03	50.9	52.427	[47]
Copper-exchanged LTA zeolite membranes	Water/ethanol	10 wt.% water, 70 °C	3.52	3591	12,640	[48]
ZSM-5-filledpolydimethylsiloxane/PES	Butanol/water	4.5 wt.% water, 31 °C, 1800 Pa	0.11	30	3.3	[49]
APTES -DD3R membrane	Water/acetic acid	10 wt.% water, 75 °C, <200 Pa	0.56*	>10,000	5600	[50]
NaYzeolite membrane	Ethanol/ethyl tert-butyl ether	20 wt.% ethanol, 60 °C	1.30	1100	1430	[51]
ZSM-5 filled PVA membrane	Water/IPA	20 wt.% water, 90 °C, 100 Pa	2.3	>100	230	[52]
ZSM-5 filled PDMS membrane	Butanol/water	1.5 wt.% butanol, 47 °C, 50 kPa	0.100	77	7.7	[53]
Hollow fiber supported NaA zeolite membrane	Water/ethanol	10 wt.% water, 75 °C	4.22	10,000	42,200	[54]
LTA membranes	Water/methanol	10 wt.% water, 50 °C, 1000 Pa	0.31*	860	266.6	[55]
PVA-NaY/PA-6 composite	Ethanol/ethyl tert-butyl ether	20 wt.% ethanol, 30 °C	-	2.3	-	[6]
PVA-NaY composite	Water/IPA	12.3 wt.% water, 35 °C, 50 Pa	0.005	2690	13.4	[56]
MFI zeolite membranes	Ethanol/water	5 wt.% ethanol, 60 °C, 50 kPa	1.85	59	109.1	[57]
MFI zeolite membranes	Ethanol/water	3 wt.% ethanol, 60 °C, 50 kPa	1.40	79	110.6	[58]
LTA membranes	Water/methanol	10 wt.% water, 60 °C, 1 kPa	0.16	10,000	1600	[37]
	Water/ethanol	9.6 wt.% water, 75 °C, 1 kPa	0.74	>100,000	74,000	
	Water/IPA	9.7 wt.% water, 75 °C, 1 kPa	1.20	>100,000	120,000	
Sandwiched (SiO_2_)/(silicalite-1)/(SiO_2_)	Ethanol/water	5 wt.% ethanol, 60 °C	2.3	136	312.8	[59]
	n-butanol/water	5 wt.% n-butanol, 60 °C	2.2	113	248.6	
MFI zeolite hollow fiber	Ethanol/water	5 wt.% ethanol, 25 °C, 100 kPa	3	160	480	[60]
NaA membrane	Water/hydrazine hydrate	20 wt.% water, 25 °C, 1333 Pa	0.064	12	0.76	[61]
NaX/ethylcellulose membrane	Water/hydrazine hydrate	20 wt.% water, 25 °C, 1333 Pa	0.012	9	0.10	
ZSM-5 membrane	Water/acetic acid	10 wt.% water, 75 °C	0.98	3200	3136	[62]
Modernite membranes	Water/acetic acid	10 wt.% water, 75 °C	0.97	1200	1164	[63]
Laterite zeolite-geopolymer membrane	Ethanol/water	8 wt.% ethanol, 70 °C	537	0.48	257.7	[64]
FER zeolite membrane	Water/ethanol	10 wt.% water, 75 °C, 6 Pa	0.045	377	16.9	[65]
Chabazite zeolite membranes	Water/ethanol	10 wt.% water, 75 °C, <200 Pa	6.25	1950	12,187	[66]
Zeolite FAU membrane	Water/DMC	10 wt.% water, 80 °C, <100 Pa	3.60	>10,000	36,000	[67]
MFI nanosheet membrane layer	Ethanol/water	40 wt.% ethanol, 60 °C	58.8	20.7	1217	[68]

* Reported as water permeate flux.

**Table 3:** Zeolite membranes studied for the separation of p-xylene/o-xylene.

Membrane	Temperature (°C)	Flux (kg m^−2^ h^−1^)	Separation Factor (β)	Reference
ZSM-5−carbon supported stainless-steel	25	0.12	1.46	[38]
Silicalite supported alumina	25	0.024	16	[73]
Oriented MFI supported alumina	25	0.15	2.3	[74]
MFI supported alumina	25	0.16	0.94	[75]
Al-ZSM-5/silicalite-1 supported stainless-steel	110	0.191	5	[76]
H-ZSM-5supported stainless-steel	100	0.027	2.29	[77]
MFI supported nanosheet	250	0.015	7700	[78]
MFI supported alumina	125	3.0	66	[79]
MFI supported alumina	26	0.050	0.18	[80]

**Table 4:** Zeolite membranes studied for pervaporative desalination.

Membrane	Operating Parameters	Flux (kg m^−2^ h^−1^)	NaCl Rejection (%)	Reference
AEL zeolite-polyamide nanocomposite	2 g L^−1^ NaCl, 25 °C	4.3	99.9	[88]
AEL zeolite-polyamide nanocomposite	36 g L^−1^ NaCl, 25 °C	3.3	99.9	
NaA zeolite membranes	35 g L^−1^ NaCl, 75 °C, <400 Pa	9.58	99.9	[90]
NaA zeolite membranes	35 g L^−1^ KCl, 75 °C, <400 Pa	8.62	99.9	
NaA zeolite membranes	35 g L^−1^ CaCl_2_, 75 °C, <400 Pa	9.35	99.9	
NaA zeolite membranes	35 g L^−1^ MgCl_2_, 75 °C, <400 Pa	8.69	99.9	

## Data Availability

Data are contained within the article.

## Abbreviations

A_m_	Active area
APTES	3-aminopropyltriethoxysilane
β	Separation factor
D	Diffusivity
DD3R	Deca-dodecasil 3 rhombohedral
DMC	Dimethyl carbonate
IPA	Isopropanol
J	Permeate flux
m_i_	Mass of compound *i*
MTBE	Methyl *tert-*butyl ether
NMP	N-methyl pyrrolidone
P	Permeability
PDMS	Polydimethylsiloxane
PEC	Positively charged polyelectrolyte
POTS	1H,1H,2H,2H-perfluoroalkyltriethoxysilanes
PSI	Pervaporation separation index
PV	Pervaporation
S	Solubility
t	Time
ZSM-5	Zeolite Socony Mobil–5
